# General Medicine and Therapeutics

**Published:** 1896-11

**Authors:** 


### GENERAL MEDICINE AND THERAPEUTICS.

### How Shall We Feed the Baby.

Dr. John T. Winter, in the *Journal of Obstetrics*, touches some disputed points in infant feeding under the above title.

"The question as to whether milk should be given fresh or boiled is one that should receive some attention. There is no question but that the heat of sterilization robs the nuclein of the milk of its vital properties to some extent, but where we are dependent on the dealer who receives his supply of milk from the country and has it in his dairy a number of hours before it is delivered to his customers, it should be either sterilized, pasteurized, or boiled; but where it can be had fresh and free from bacteria, it can be used in the raw state and can be warmed sufficiently by the water with which it is diluted, and it is thought in this way to be more nourishing and more easily digested.

"I do not think it wise to habitually peptonize milk for healthy children, as their digestive organs, not being called on for full duty, are, I fear, weakened; and the careless manner in which the sterilizing of milk is frequently done makes it many times rather harmful than beneficial, as it makes the coagula of casein in cow's milk much harder to digest.

"My objection to the use of condensed milk is that while the infant will fatten on its use, it is usually found to be pale and flabby, and does not, many times, have strength enough to carry it through childhood. By the addition of cream and the water from barley or rice, or a thin oatmeal gruel, we can improve it in this particular.

"During the first week or two after birth the child should be fed every two or three hours, and will require about one ounce at each feeding, about half a pint a day—good milk, two drachms; boiled water, rice water, or barley water, six drachms.

"By the second week, and up to about the sixth week, it will require about one pint a day, and, unless the milk is first-class, it will be well to add sugar-of-milk and cream—sugar-of-milk, four grains; cream, one drachm; milk, four drachms; water, one and a half ounces.

"At from about the third to the sixth month it will take nearly four ounces at each meal, nearly a quart a day—sugar of milk, one scruple; cream, two drachms; milk, one ounce; water, two ounces.

"From this time on about the same proportions should be maintained, the quantity being gradually increased until three pints—perhaps a little more—are taken in twenty-four hours, the quantity of water being gradually reduced, or a thin oatmeal gruel can be substituted for the rice or barley water. A small pinch of salt can occasionally be added. This gruel can be gradually thickened, so that by the time the child is ten or twelve months old it can have, say, two meals a day of oatmeal gruel and three of pure cow's milk.

"It is better for the child up to this time to keep it from the table and not to allow friends or strangers to give it candy, cakes, or other food, but from this time on potatoes and other suitable articles of diet can be added."—*American Medical Review.*

---

### CONSUMPTION AMONG THE COLORED POPULATION OF THE SOUTHERN STATES.

Hubbard (Tennessee Medical Society, April 14) says: As far as I have been able to ascertain, from the testimony of many physicians who practiced in the South before the late civil war, pulmonary consumption was a comparatively rare disease among the slave population, some even affirming that it was entirely unknown.

According to the United States census of 1890 the total number of deaths from consumption in the United States, for the year ending May 31, 1890, was 102,199; of these 84,173 were white, and

18,026 were colored; the per cent. of deaths from this disease being 11.1 among the white population and 15.4 among the colored. In the State of Alabama for the same year the proportion 8.1 white, 12.7 colored, or about fifty per cent. greater.

Recent statistics from Atlanta, Ga., Baltimore, Md., Charleston, S. C., Memphis, Tenn., Nashville, Tenn., Richmond, Va., Macon, Ga., St. Louis, Mo., show that the average proportion of deaths from consumption to that of total mortality is, 10.4 of the white population, to 16 of the colored; or nearly three times as great.

It is generally believed that those of pure African descent are less susceptible to this disease than those of mixed blood.

Among the most important causes of this excessive mortality are the following:

1. Ignorance concerning the laws of hygiene.
2. Unhealthy dwellings, often situated on damp and narrow alleys, reeking with filth.
3. Improper food, lacking in quantity and of poor quality.
4. Insufficient clothing and exposure in inclement weather.
5. Irregular habits and lack of proper amount of sleep.
6. Excessive use of intoxicating drink and other vices.
7. Lack of medical attention and proper nursing.

As remedies I would suggest:

1. Thorough instruction in practical hygiene in all colored schools and higher institutions of learning.
2. Prohibition by law of the use of dwellings, school buildings, and churches unfit for habitation; and frequent examinations by competent sanitary inspectors.
3. The sale of adulterated and unhealthy food should not be allowed, especially milk from cows affected with tuberculosis.
4. The use of intoxicating liquors, except as medicine, should be prohibited.
5. Those who are unable to procure proper medical attention should be provided with physicians at the public expense, and hospitals for consumptive patients should be prepared for the reception of those who are affected with this disease.
6. All cases of consumption should be promptly reported to the proper health officer, who should give the necessary instruction as to the best sanitary measures to be pursued; and after the death of the patient the premises ought to be properly disinfected as is done in the case of other contagious diseases.—*Charlotte Med. Jour.*

---

### SNAKE-VENOM ANTITOXIN.

Mr. E. H. Hankin states that the poison secreted by the microbes of tetanus and diphtheria are closely allied to, if not indistinguishable, chemically, from the poison secreted by venomous snakes. Mitchell and Wolfenden have discovered that the venom of snakes owes its properties to the presence of a poisonous albumin, that of the cobra, according to the author, being so active that for the mouse the lethal dose of the dried poison has been found to be no more than the thirteen-millionth part of its body-weight. Sewell showed in 1887 that it was possible to make pigeons immune against rattlesnake-venom by injecting into them a series of minute doses of this substance. The question of the possibility of producing antitoxins which might neutralize the poison of venomous snakes has been taken up by Calmette and Fraser. Both have succeeded in producing a high degree of immunity against snake-poison in animals, and both have found that serum of such animals possesses the power of neutralizing cobra venom. The former claims that the activity of his antivenine is ten thousand units. There is little doubt that antivenine would produce immunity in a safe and easy way if injected an hour previous to the bite, and in a small dose. While it is easy to produce an immediate though temporary immunity, it is comparatively difficult to effect a cure when once the symptoms have arisen. Hence the one hope of success is to inject the remedy at a sufficiently early date after the bite. The limb should be well ligatured, in hope that the remedy may be absorbed into the system in advance of the poison. Since death within half an hour after the bite of a venomous snake is the exception, and since, as a rule, more than three hours elapse before the fatal issue, and, further, that antivenine, even when used an hour and a half after the bite of extremely venomous snakes, has been found to be efficacious, there is good reason for expecting favorable results from this treatment, which has been found to be active against the poison of all species of snakes in which it has hitherto been tried. The injection is made, under antiseptic precautions, into the subcutaneous cellular tissue of the flank. Then with the same syringe injections of aqueous solutions of lime chloride (1 to 60) are made about the bite, which has been previously washed with the same solution. Neither alcohol nor ammonia should be administered, since these may interfere with the action of the remedy.—*Indian Medical Gazette*, 1896, No. 2, p. 56- *Quoted in Am. Jour. Med. Science*, May, 1896.

---

### Treatment of Tetanus.

Trevelyan (*British Medical Journal*, February 8, 1896) reports a case of cephalic tetanus treated by injections of antitoxic serum. The serum was obtained from Roux, of Paris. Half of the serum in the tube was injected on the second day and the other half on the third day, but no trace of improvement could be detected even by the most interested witnesses. The author states that it is the third case in which he has assisted in the serum treatment, and in no case did it exercise the slightest influence on the course of the disease. There were in all seven cases.

Willard and Johnson (*Transactions of the Philadelphia College of Physicians*, vol. xvii, p. 27) reported a case of recovery from cephalic tetanus following a slight injury in the neighborhood of the eye. The treatment consisted in loosening the scar and administering sedatives. The patient was a boy twelve years of age, and the doses were increased until he took, in each twenty-four hours, seventy-five grains of bromide of potassium, thirty grains of chloral, and thirty-six drops of the deodorized tincture of opium.

Dr. Delbecq, in the *Nord Médical*, reports a case of a boy thirteen whom he cured of tetanus, following in some twelve hours after a wound of the knee, produced when bathing by a broken vase. Bromide and chloral, and later chloral and morphia, were given and stopped on the second day. It required three 10.c.c. doses of the "anti-tetanique" to turn the balance in favor of recovery.

### SOME THERAPEUTIC USES OF GUAIACOL.*

I desire to give my experience, gleaned from bedside observation, of that little-written-about drug, guaiacol.

Its use as a local application for the reduction of temperature was first suggested to me by Dr. J. Solis-Cohen, some two years ago, in a case of tuberculosis which he saw in consultation with me. Its action in this case was so prompt and effective that I pushed the investigation in other diseases, with results herewith summarized.

In September, 1894, when I entered upon my term of service in the Williamsport Hospital, I commenced the use of guaiacol as a local application for the reduction of temperature. In typhoid fever, since then, I have had it applied 864 times—778 times in the hospital, and 86 times in private practice. These applications were made to forty-three different persons, about equally divided between males and females, with wide variation of ages. The greatest number of times it was applied to any single person was 78, and the least number of times, once. The largest dose was twenty-five drops and the smallest two drops. The greatest reduction of temperature was from 106.8° to 101°, in two hours, by the application of five drops, with a corresponding reduction of the pulse-rate from 136 to 110 per minute, the respirations falling from 36 per minute to 28. This patient, however, showed very great susceptibility to the drug, as the application of two drops reduced the temperature from 103° to 100.4° in one and one-half hours. A number of special reports were prepared for me by the nurses, of some of the cases in which guaiacol was applied, which may be useful in showing its effect not only upon the temperature but also upon the pulse:

*Carrie Horton :*

| Five drops of guaiacol applied. | Pulse. | Temp. |
|---|---|---|
| 9:45 P.M. | 144 | 107° |
| 10:20 P.M. | 132 | 104.2° |
| 10:50 P.M. | 132 | 102.2° |

* Read before the Philadelphia County Medical Society, April 22, 1896, by Horace G. McCormick, M.D., Williamsport, Pa., Physician to the Williamsport Hospital, Pennsylvania.

| Five drops applied. | Pulse. | Temp. |
|---|---|---|
| 12:45 P.M. | 130 | 104° |
| 1:30 P.M. | 120 | 100.6° |

| Five drops applied. | Pulse. | Temp. |
|---|---|---|
| 5 A.M. | 118 | 104° |
| 5:30 A.M. | 108 | 102.4° |

| Five drops applied. | Pulse. | Temp. |
|---|---|---|
| 1:25 P.M | 135 | 104° |
| 2:20 P.M. | 130 | 101.8° |

*Ada Saxton:*

| Twenty drops applied. | Pulse. | Temp. |
|---|---|---|
| 11 A.M. | 126 | 104° |
| 12:30 P.M. | 112 | 101° |
| 3 P.M. | 110 | 100° |
| 4 P.M. | 122 | 99° |

*Annie Witzman:*

| Ten drops applied. | Pulse. | Temp. |
|---|---|---|
| 10 P.M. | 130 | 104.4° |
| 11 P.M. | 108 | 100.6° |
| 12 midnight | 105 | 98.2° |
| 12:30 A.M., slight chill. | | |

| Fifteen drops applied. | Pulse. | Temp. |
|---|---|---|
| 12 M. | 126 | 105.2° |
| 1 P.M. | 120 | 101.4° |
| 2 P.M. | 106 | 100.8° |

| Fifteen drops applied. | Pulse. | Temp. |
|---|---|---|
| 10:40 P.M. | 156 | 107° |
| 11:15 P.M. | 134 | 104° |
| 12 midnight | 116 | 102.4° |
| 12:45 A.M. | 108 | 99.8° |

It will be noticed that within thirty minutes after the application there was a fall in the temperature, and in most cases a corresponding reduction of the number of heart-pulsations per minute. It is generally asserted that guaiacol is a depressant, and for this reason, a dangerous remedy in diseases in which the circulation is likely to suffer from long-continued fever. This assertion has not been borne out by my experience. On the contrary, I have seen at different times, with a high temperature, a pulse so rapid and weak that it could not be accurately counted, after the application of guaiacol distinctly lessened in frequency and strengthened in force. A weak and rapid pulse is to me no contraindication for the use of the drug.

In the case of Annie Witzman the reduction of temperature is well illustrated; seventy-eight applications were made. It will be noticed that on November 11th, at 10:40 P.M., the temperature reached 107° and the pulse was 156. Fifteen drops of guaiacol were then applied, and at 12:45 P.M. the temperature was reduced to 99.8° and the pulse was 108. The table does not show this. The ice-pack was used in this case, being kept up for twelve consecutive hours; yet I was forced to abandon this and return to the guaiacol in order to reduce the temperature. This case was one of the most persistent cases of high temperature I have ever seen. The patient made a good recovery.

The effect of guaiacol lasts from three to four hours; the more often it is applied the greater the effect. When I first commenced the use of this drug I found that the sudden reduction of temperature caused chilling in a number of cases, but after I became more accustomed to its use chills rarely occurred. If it can be avoided, the temperature should not be reduced below 100°; and this is a matter which can easily be regulated after the applications have been made a few times, care being taken to commence with a small dose—say from ten to fifteen drops—this gradually being increased if necessary until the temperature is reduced.

It has been suggested that by this rapid reduction of temperature there is great danger of producing congestion of some of the internal organs of the body. I have not seen a single unfavorable symptom (except an occasional chill) following its use. I had one case of pneumonia as a complication, but this did not develop until six days after the last application of guaiacol, which was in no way responsible for it.

The point selected for the application was the right iliac region. This was thoroughly cleansed with soap and water, and after the part was thoroughly dried (it is important that the surface should be entirely free from moisture, for guaiacol being of an oily nature will not be absorbed if there is the least moisture present) the guaiacol was slowly dropped upon the surface and thoroughly rubbed in with the hand for from ten to fifteen minutes. The part was then covered with oiled silk or waxed paper. The only preparation used was Merck's, and it rarely failed to produce the desired result. Any other point would probably do as well for the application, but I selected this because it was as near the seat of the trouble as I could possibly get, and could be easily reached and covered with the oiled silk, without in any way disturbing the patient. In only three cases was any local irritation produced, and I was forced to move to the left side to make my applications.

There were no unpleasant symptoms accompanying its use, and no complaint was made by any of the patients. The disagreeable odor that has been described by some as being objectionable, was referred to by only one of my patients, and then only on the first application. Sweating is nearly always produced, corresponding somewhat to the greater or less reduction of temperature.

One very important fact was observed in the use of this drug for the reduction of temperature in a case of pyemia. The case was seen in consultation with Dr. Detwiler, and was found with a temperature of 107°. Twenty drops of guaiacol produced no effect. The application was repeated in forty minutes, and yet no effect on the temperature was produced. Fifty minutes later another application (this time forty drops) was made with the same result as before. The applications were continued until the expiration of three hours, when 100 drops had been applied; yet during this time the temperature had not varied over half a degree from 107°. The pulse was weak and rapid when the first application was made, at the end of three hours had lessened in the number of beats per minute and increased in volume. There was no sweating in this case. I have not had an opportunity of trying guaiacol in any other case of pyemia, but from my experience in this one I am led to believe that it has no value.

It may be urged that the fever of typhoid is not the disease, and does not call for treatment. That it is the disease no one will pretend to assert; but that it is an important symptom, and when it rises beyond a certain point calls for something to reduce it, or keep it in abeyance, every physician who has had much to do with this disease can bear testimony.

When the system of baths was first introduced, the only object was the reduction of temperature, but those using them found that their patients did so well under this form of treatment that they took to philosophizing upon the subject, and undertook to prove that the cold bath did something else not exactly admitting of explanation, by modifying the disease, lessening the fever, and making the case a less formidable one. That this effect is produced I am willing to concede, but that the baths do more I believe is open to serious question, and further proof will be required to place it beyond controversy in the profession.

Much has been written by the advocates of the system of the delight and pleasure their patients have experienced in being put in a cold bath. I believe these gentlemen have never tried it themselves. I have no reason to believe that the people of Philadelphia or Baltimore are any less susceptible to cold than those in the interior of Pennsylvania. I have used the cold bath many times, and, with the exception of a colored boy whom I treated in this way some time ago, I have not been able to make my patients believe by any argument of mine that it was an enjoyable pastime. I thoroughly believe in it as an effective form of treatment, but it is not pleasant for the patient; it is difficult to carry out in detail; it involves great expense, and, in private practice, is not a practical form of treatment. In spite of the invention of portable bathtubs, the method still lacks that practicability that is demanded by the general practitioner.

Guaiacol, when given internally, does not markedly reduce the temperature. I have given as high as ten drops in a single dose to a patient without materially affecting the pyrexia, while the same amount thoroughly applied to the skin would reduce the temperature to a marked degree. I have also applied the remedy to those in health in sufficient doses to reduce an abnormal temperature, without any effect upon either the temperature or the pulse.

The natural question to ask would be: If this drug possesses the power as here asserted, what is its method of action? How does it reduce temperature? What is the physiologic action? Now, in order that there may be no controversy, with me at least, upon this subject, I candidly assure you that I do not know. I know the effect, but cannot explain how it is brought about.

While I have a high regard for guaiacol as a local antithermic, none of this respect is lost when I come to use it internally. As an intestinal antiseptic, I believe it has no superior. I have used it in fifty-six cases of typhoid fever, with the best results. The best evidence of its virtue in these cases is the fact that tympanites was either entirely abolished or was present in so slight a degree as to be of no consequence. The tongue was moist throughout the disease, and delirium, if present, was of a very mild form. Of the fifty-six cases thus treated, fifty-five recovered. In the one in which death took place, the patient was admitted to the hospital on the fourteenth day of his sickness, and died on the nineteenth—of perforation; an autopsy was made, and it was found that the perforation had taken place about three inches above the ileo-cecal valve. When the abdomen was opened, the unmistakable odor of guaiacol was found to be present. This was to me an important discovery, as by the death of this man was proved—what I had before been led to believe—that guaiacol passes through the alimentary canal unchanged, for the guaiacol which had been given this man was still guaiacol at the ileo-cecal valve, and passage through the colon would not be likely to change it. The smell of guaiacol was noticed in the stools of nearly all of these patients. It has been my practice to give pure guaiacol in emulsion. This is rather a disagreeable dose, and some objected so seriously to its use that I was forced to give it in capsules; when this was not expedient, then I gave guaiacol of carbonate dry upon the tongue in two and a half grain doses. I have never found this to disagree with the stomach, and patients do not offer any objection to taking it.

In summing up this question, I feel convinced of the following facts:

That guaiacol, when applied locally in typhoid fever, is certain to reduce the temperature.

That, with the care that a physician should always use in the administration of drugs, it is absolutely safe.

That chills will not occur if the temperature is not reduced below 100°.

That no deleterious effects are produced upon any organs of the body by its use.

That it is easy to apply, and can be used by any one competent to nurse a typhoid-fever patient.

That there are no depressing effects following the use of the drug, if used intelligently.

That by its continued use the dose can be gradually lessened.

That it is far superior to the cold bath, in that it can be used by one person. No appliances are necessary for its use that are not obtainable in any house. It is much more pleasant to the patient, and carries with it no horror to the family. Argument and persuasion with the family and friends are not made necessary. It is fully as effective as the cold bath. Patients are not subjected to the danger of moving. There is no complication, such as hemorrhage, etc., that is a contraindication to its use.

That when given internally it is one of the best intestinal antiseptics known, if not the best. By its use in typhoid fever, the dry tongue and tympanites are abolished, digestion and assimilation rendered more perfect, and the probabilities of hemorrhage reduced to almost *nil*.—*Medical and Surgical Reporter*.

